# Green processing protocol for germinating and wet milling brown rice for beverage formulations: Sprouting, milling and gelatinization effects

**DOI:** 10.1002/fsn3.1534

**Published:** 2020-04-13

**Authors:** John C. Beaulieu, Shawndrika S. Reed, Javier M. Obando‐Ulloa, Anna M. McClung

**Affiliations:** ^1^ United States Department of Agriculture Agricultural Research Service Southern Regional Research Center New Orleans Louisiana; ^2^ Doctorate Program on Natural Science for the Development and Agronomy Engineering School Technology Institute of Costa Rica (ITCR) San Carlos Technology Local Campus Alajuela Ciudad Quesada Costa Rica; ^3^ United States Department of Agriculture Agricultural Research Service Dale Bumpers National Rice Research Center Stuttgart Arkansas

**Keywords:** enzymes, green processing, rapid visco analyzer, starch, sugar, viscosity

## Abstract

Rice‐derived beverages offer a non‐soy, lactose‐free, cholesterol and gluten‐free food source, which may offer well‐balanced nutrition. Brown rice is nutritionally superior to white rice but oil oxidation and rancidity can be problematic regarding organoleptics during processing and storage. Using green technologies, which do not rely upon stabilization, brown rice was sprouted and processed with enzymes to produce preliminary value‐added rice beverages. Paddy (rough) Rondo rice was dehulled using a pilot plant dehusker, sorted and cleaned into brown rice (BRR), rinsed, and germinated under various conditions (times and temperatures). Germinated brown rice (GBR) was then assessed (96.7 ± 0.8% germination and coleoptile length 2.24 ± 0.83 mm) prior to developing a method to soften, wet mill, sieve and gelatinize the matrix. Moderate macronutrient catabolism based on proximate analysis (e.g., 27.0%, 30.9% and 28.9% protein, oil and carbohydrate loss, respectively) and significantly decreased phytic acid (71.6%) from BRR → GBR along with processing efficiency were used to establish a germination and processing protocol engaging the natural enzymatic hydrolysis of starch and other biochemical changes. Based on rapid visco analyzer pasting properties in heated BRR, GBR sieving results and observations of stored crude beverages, proteins and oils apparently remained soluble and were conveyed forward into an the enzyme‐treated solubilized oligosaccharide matrix, which could be a natural emulsion. A method for germinating and processing brown rice, leading to a completely green process and “free‐flowing” soluble matrix to deliver preliminary sprouted brown rice beverages is presented.

## Graphical abstract

Using green technologies which do not rely upon stabilization, brown rice was germinated, thermally softened, wet‐milled, gelatinized and processed with enzymes to produce value‐added sprouted brown rice beverages. A “free‐flowing” process maintains endogenous components in germinated brown rice through a soluble matrix, requiring no stabilization, oils, emulsifiers, and to date, no fortification or additives. The only exogenous additions were food‐grade enzymes that were utilized for liquefaction. Herein, the process is detailed along with proximate analyses of white, brown and germinated brown Rondo rice, microbial assessment, pH, color and soluble solids of the developed beverages, and demonstrating the process decreased significantly the anti‐nutrient phytic acid in the germinated brown rice.

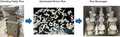

## 1 INTRODUCTION

U.S. estimated rice crop in 2018 was valued at 2,752 million dollars (USDA, 2019a). World production of milled rice has been around 480 million metric tons (MMT) since 2016 (USDA, 2018). Meanwhile, U.S. rice consumption (and residuals) has remained somewhat constant around 4.06–4.23 MMT over the last three years (USDA, 2018). Storage, milling and further food processing affect lipids, starch and protein, resulting in sensory and textural changes of final rice products in the marketplace. The vast majority of rice nutrients are concentrated in the bran fraction, including the oil with essential fatty acids, proteins, fiber, vitamins, antioxidants and other micronutrients (Champagne, 2004; Juliano, 1985; Saunders, 1990). Subsequently, brown rice containing bran, embryo, and aleurone convey health‐promoting nutritional constituents (Cho & Lim, 2016; Han, Arijaje, Jinn, Mauromoustakos, & Wang, 2016; Kim et al., 2012; Wu, Yang, Toure, Jin, & Xu, 2013), offering superior health‐benefits for consumers.

Globally, consumers are concerned about saturated fat levels in foods, lactose intolerance, hormone and antibiotic levels in dairy products, as well as sustainable treatment of the environment and animal food production. The burgeoning functional beverage market (AIJN, 2018; Moloughney, 2016) offers a consumer‐friendly mechanism to provide healthy alternates to other beverages. Thus, development of improved sprouted brown rice beverages offers new market opportunities that address many of these concerns.

New uses of rice in value‐added food products are being explored because rice is a lactose‐free, cholesterol and gluten‐free food. By 2011, U.S. retail sales of plant‐based nondairy beverages (almond, coconut, hemp, rice and soy milk) attained $1.3 billion (Kadey, 2012), and were projected to reach $2.9 billion by 2017 (Sloan, 2014). “Worldwide sales of nondairy milk alternatives more than doubled between 2009 and 2015 to $21 billion, according to Euromonitor” (Whipp & Daneshkhu, 2016). Meanwhile, per capita fluid dairy milk consumption has steadily declined in the U.S., dropping 40.9% from 1975 to 2018, according to the U.S. Department of Agriculture (USDA, 2019b).

Several patents and methods are available for making rice syrup or beverages from stabilized rice bran (Hammond, 1994), rice flour (Servotte, 2008), rice (whole, brown, white) (Bartocci & De Luigi, 2000; Mitchell & Mitchell, 2010; Mitchell, Mitchell, & Nissenbaum, 1988, 1990; Ravagnani & Sambataro, 2004), preroasted rice (Nam, Seo, Kim, & Kim, 2001), pregerminated brown rice (Mei, 2014) and a rice slurry (Koyama & Kitamura, 2014; Ravagnani & Sambataro, 2004). Wet milling and/or soaking (Chiang & Yeh, 2002; Koyama & Kitamura, 2014; Ravagnani & Sambataro, 2004), along with enzymes (principally α‐amylase and glucoamylase), emulsifying substances and homogenization are common practices utilized in rice beverage production (Bartocci & De Luigi, 2000; Mei, 2014; Mitchell & Mitchell, 2010; Ravagnani & Sambataro, 2004). Aside from one patent (Mitchell et al., 1988) and revision thereof (Mitchell et al., 1990), scant literature was found that clearly indicates processes for producing rice beverages from whole grain sprouted brown rice that was not previously processed, stabilized or defatted.

The use of stabilized dehulled whole grain brown rice and bran or residual press cakes indicates that costly and/or chemical treatments have likely been used to deliver shelf‐stable commercial products. Various extraction methods and proven extraction techniques are no longer favored by many manufacturers and consumers who desire less processed, healthier, “green” products. “Green technologies” for food processing is defined herein as sustainable, less harmful to the environment, and safe natural chemical processes used to transform raw products into value‐added foods and ingredients, including use of endogenous and food‐grade enzymes which, provide reaction specificity, sensitivity and nontoxicity. Commercially available, truly natural, nonfortified, unflavored nondairy rice beverages are scarce. Recently there has been a re‐invigorated health trend using sprouted whole grain products (bread flours, cereals and beverages) which have markedly increased in the food and beverage industry marketplace (Pagand, Heirbaut, Pierre, & Pareyt, 2017). Germination (sprouting) is a low‐cost technology that starts with seed water uptake (imbibition) and ends with the protrusion of the radicle from the seed. Activation of seed metabolism occurs during the germination process, which results in the hydrolysis of storage proteins and carbohydrates and the synthesis/accumulation of metabolites with health‐promoting properties (Wu et al., 2013). Sprouts and microgreens for direct human consumption also fall under strict production regulations (FDA, 2015; SSA, 2017).

By 2018, the number of rice beverages on the market had dramatically dropped compared to four to five years ago, and this product category was being replaced by alternate plant sources, and pregerminated or sprouted seeds, which naturally elevate health‐promoting components. Yet, many products often contain fillers, flavors, or additives (whey, honey, other seed oils, carrageenan, gellan gum, xanthan gum, seaweed, vanilla, etc.) that have utility regarding fortification and chemistry for stability, mouthfeel, viscosity, etc. Many of these rapidly advanced products appearing on grocer's shelves have not been well characterized and reported in scientific literature. We have developed methods to deliver superior all‐natural value‐added rice beverages using green technologies. Our goals are to outline germination parameters, softening, and wet‐milling protocols and enzymatic treatments to deliver sprouted brown rice beverages. Sprout characteristics, microbial considerations, proximate analyses, processing loss, brown versus white rice viscometry, and rapid beverage quality assessments are reported.

## 2 MATERIALS AND METHODS

### 2.1 Rice source: rondo milled and brown rice

Breeder seed stock of the rice variety Rondo (PI 657830) was grown at the Dale Bumpers National Rice Research Center in Stuttgart, Arkansas, in 2014 using standard production practices. The variety was drill seeded on April 22, 2014, and emerged three weeks later. On June 4, 112 kg N/ha (urea) was applied, and the field was then irrigated and remained flooded until about 10 d prior to harvest on September 18. The grain was harvested at approximately 18% moisture and then dried to 12% using a forced air drier. Rough rice was cleaned using a screen cleaner (Model MICRO‐224‐LH, Crippen Northland Superior Supply Co.) and stored at 4°C at 60% relative humidity for two months prior to shipment to the Southern Regional Research Center (SRRC) in New Orleans, Louisiana. Using the same seed lot, approximately 23 kg of milled white rice (WR) was produced by the USDA in Stuttgart, AR. Dried rough rice was dehulled using a Yamamoto Impeller Type Husker (Model FC2K, Calibration Plus) and milled rice was produced using a Yamamoto Miller Rice Pal (Model VP‐32T, Calibration Plus) with the whitening adjustment set at 5. The rice was not separated but shipped as whole milled rice and brokens to the SRRC in New Orleans, Louisiana.

Paddy and milled rice were stored at 5°C until use. Paddy rice was dehulled (Satake Husk Aspirator, HA 60B) at the SRRC and then manually sorted through three stacked standard sieves (U.S. #6, #7, and #8, 3.36–2.38 mm, Gilson Co., Inc.) followed by removal of off‐colored or insect‐damaged kernels, or sorted and graded using a Clipper 400 Office Tester Cleaner (A. T. Ferrell Company). The Clipper was used with an 11R (round) top screen and a 10 × 10 wire (square wire mesh) bottom screen with full ventilation blower, and kernels were passed twice. Percentage product weight of brown Rondo rice (BRR) and dehulling and culling loss were monitored.

### 2.2 Optimization of germination protocol for beverage formulation

Twenty‐five g (DW) of paddy Rondo rice and freshly dehulled (BRR) were soaked in 75 g deionized water housed in 500‐ml glass jars receiving a flow‐through of breathable air using a bubbling stone at 100 ml/min flow rate with various durations (48 to 72 hr) and temperatures (20, 30, and 35°C). Weight change and coleoptile length were monitored through 72 hr. Sprouting was terminated when approximately 2–4 mm shoots had protruded and the vast majority of kernels had germinated. These tests compared germination with and without hulls since methods in the patents and literature are oftentimes difficult to decipher if hulls were removed. Ultimately, only dehulled materials were chosen and designated as germinated brown rice (GBR).

Percentage germination was measured in randomly selected and counted piles of dehulled rice, always exceeding 100 kernels. Broken kernels randomly selected were as follows: (a) counted if the embryo side was present, or (b) discarded and not used in the tally when the piece was from the kernel apex. An electronic Vermeer caliper (Westward) was used to measure coleoptile (plumule) length in randomly accrued piles of germinated kernels whereby only severely curved coleoptiles were not tallied.

**Figure 1:**
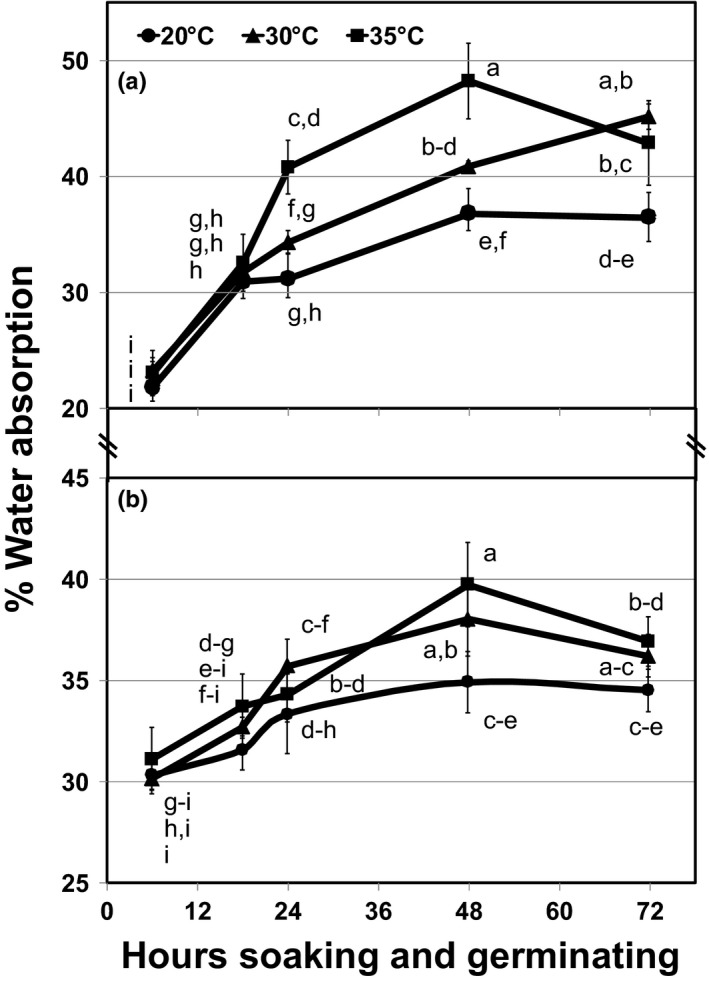
Water absorption (% weight change) during soaking and germination in (a) Rondo paddy rice and (b) freshly dehulled brown Rondo rice (BRR) at various temperatures. Means not connected by same letter, per panel, are significantly different according to a Tukey–Kramer HSD at *p* < .05

After initial assessments (above), freshly dehulled BRR was used immediately or within 24 hr (Mitchell & Mitchell, 2010) for sprouting. After rinsing using a flour sifter wire mesh screen to remove residual dehulling debris, BRR was presoaked then germinated for 48 hr at 35°C, generating GBR. An optimum time and temperature was selected based on Figure 1, as bracketed by proximate evaluations reported in Table 1, and 400 or 600 g (DW) freshly dehulled BRR was sprouted in the dark in glass mason jars fitted with sprouting screen caps. All BRR was soaked/rinsed for 30 min, prior to 1.5× volume of added deionized 35°C water, exchanged on 4 hr cycles. Every 4 hr, jars were carefully swirled, water decanted, rinsed three times, and fresh water added. Thereafter, sprouting occurred within the jars held upside down, with thorough rinsing occurring every 4 hr, at 35°C (Table 2).

**Table 1:** Proximate analyses and specific comparisons for Rondo white, brown rice (BRR), and germinated brown rice (GBR) samples

Sample	Moisture (g/100 g)	Crude protein (%)	Crude fat (%)	Crude fiber (%)	Ash (g/100 g)	Carbs (g/100 g)	Phytic acid (%)
Paddy^a^	(14.0)	5.8–7.7	1.5–2.3	7.2–10.4	2.9–5.2	64–73	0.18–0.21
White^a^	(14.0)	6.3–7.1	0.3–0.5	0.2–0.5	0.3–0.8	77–89	0.02–0.07
White^b^	11.62	7.13	0.66	1.3	–	79.95	–
WR Rondo^c^	11.49 ± 0.03a^d^	6.60 ± 0.04b	1.19 ± 0.06b	0.83 ± 0.12a	0.86 ± 0.01b	79.03 ± 0.09a	0.37 ± 0.01b
Brown^a^	(14.0)	7.1–8.3	1.6–2.8	0.6–1.0	1.0–1.5	73–87	0.13–0.27
Brown^b^	11.80	7.54	3.20	3.6	–	76.25	–
BRR Rondo	11.97 ± 1.74a	7.41 ± 0.25a	3.59 ± 0.50a	1.39 ± 0.61a	1.38 ± 0.03a	74.25 ± 1.08b	0.81 ± 0.07a
GBR Rondo	37.08 ± 0.57*	5.41 ± 0.17*	2.48 ± 0.12*	1.43 ± 0.37	0.81 ± 0.02*	52.78 ± 0.39*	0.23 ± 0.06*

^a^ Proximate analyses as reported for stored rice held at constant 14% moisture content, FAO, Tables 14 and 15, (http://www.fao.org/docrep/t0567e/T0567E08.htm).

^b^ USDA Nutrient Database for Standard Reference Legacy Release (NDB # 20044. white, long grain, regular, raw, unenriched. NDB # 20036, brown, long grain, raw (includes foods for USDA's Food Distribution Program), (https://ndb.nal.usda.gov/ndb/).

^c^ Milled, polished breeders seed (Rondo) white rice (WR) obtained from USDA ARS, Stuttgart, AR; brown Rondo rice (BRR) was dehulled (at the Southern Regional Research Center) and germinated, GBR.

^d^ Means highlighted with an asterisk (*) are significantly different from the BRR according to Dunnett's test at *p* < .05. Means not connected by same letter are significantly different according to a Tukey–Kramer HSD at *p* < .05.

**Table 2:** Optimized free‐flowing processing parameters for preparing freshly dehulled sprouted, softened, wet‐milled, gelatinized, and enzyme‐treated brown rice beverages

Processing steps	Treatment code	Conditions	Optimized method
Controls	WR or BRR^a^	White rice, or freshly dehulled brown Rondo rice	Not applicable
Rinsing	Rinses^b^	Ambient temperature, or designated per below	30 min
Soaking		Temperature	35°C
		Temperature of added H_2_0	35°C
		Time	24 hr
		Ratio (rice:water, g/g)	1:1
		Rinsed, replaced	Every 4 hr
Sprouting	GBR	Temperature	35°C
		Rinsed	Every 4 hr
		Temperature rinse H_2_0	35°C
		Time	24 hr
Softening	(none)	Temperature	70°C max
		Temperature of added H_2_0	<75°C
		Times^c^	60 to 120 min
		Ratio (rice:water, wt/wt)^d^	1:2
Wet milling	PWM	Temperature	~65°C (70°C max)
		Temperature of added H_2_0	75°C
		Milling time	2 min
		Sum ratio (rice:water)	1:4
Gelatinization	Gelat	Temperature	80°C
Enzymes and dosage	PNZ	α‐amylase	300 µl/100 g starch
		Glucoamylase	300 µl/100 g starch

^a^ Acronyms for treatments are as follows: BRR, brown Rondo rice; GBR, germinated brown rice; PNZ, post saccharification enzymes; PWM, postwet milling and WR, white rice.

^b^ Rinses included as follows: Water rinse (Rinse); 30 and 300 ppm peracetic acid.

^c^ Trial times between 60 and 120 min due to differences in volumes, beaker sizes, number of units run simultaneously, and differences in heat energy transfer to soften kernels, as based upon a subjective softness test with a stainless steel spatula on a stainless steel table.

^d^ Considered on a dry–wet (DW) weight basis even though kernels had absorbed water weight. Based upon original grams rice to grams water utilized.

### 2.3 Assessment of microbial contamination following germination

Commercial‐like antimicrobial food‐safety rinsing treatments were compared against a deionized water rinse control. BRR was subjected to a 30 min static hold in 35°C deionized water, or 30 and 300 ppm peracetic acid (purum, Sigma‐Aldrich), followed by several flushes of deionized water rinses. Thereafter, samples were soaked and sprouted, per Table 2. A microbial appraisal in controls, rinsed kernels, and postgerminated sprouts was conducted by assessing total aerobic plate count (TPC), per the AOAC International (Association of Official Analytical Chemists) Official Method 966.23 (AOAC, 2016), and yeast and molds were enumerated according to the FDA BAM method (Tournas, Stack, Mislivec, Koch, & Bandler, 2001).

### 2.4 Proximate analysis and phytic acid (%)

Moisture Content (g/100 g) was determined after holding samples in a vacuum oven at 70°C for 6 hr. Crude protein (%) was analyzed by combustion per American Association of Cereal Chemists International, method 46–30.01 (AACCI, 1999a) where percent crude protein = % nitrogen × 5.70. Crude fat (%) was determined by acid hydrolysis per the Association of Analytical Communities, AOAC 922.06 (AOAC, 2006). Crude fiber (%) was determined after digesting with 0.127 mol/L H2SO4 and 0.313 mol/L NaOH by the filter bag technique, according to the American Oil Chemists' Society method Ba 6a‐05 (AOCS, 2017). Ash (g/100 g) content in flours was performed by the AOAC direct method 923.03 (32.1.05) (AOAC, 2000). Carbohydrates were deduced by subtraction of the aforementioned components. Phytic acid (%) was measured using the ferric ion precipitate method with inorganic phosphate (Ellis, Morris, & Philpot, 1977).

### 2.5 Thermal softening, wet milling, and size reduction

**Figure 2:**
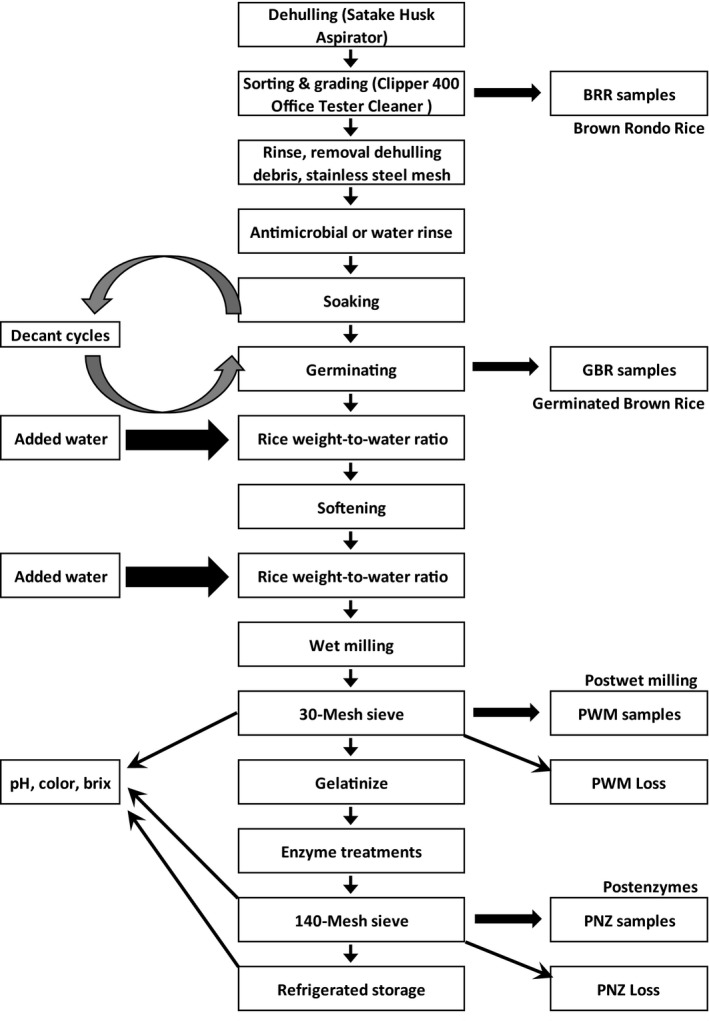
Empirically deduced optimized germinated brown rice “free‐flowing” processing protocol and sampling regime

Through several preliminary tests, an optimized protocol was deduced whereby the GBR responded consistently (softened, wet milled, and maintained a “free‐flowing” state) over several trials and established a baseline method (Table 2). Several different treatment regimens aimed toward fully softening the individual kernels were applied, attempting to minimize gelatinization during the initial stages of sieving and wet milling. Albeit subjective, a test was applied whereby a stainless steel spatula was forced upon rice kernels laid on a stainless steel bench to verify no grittiness, and a soft yield occurred. Samples were presoftened thermally, followed by wet milling in a 4‐L commercial Waring blender (CB15V, Torrington, CN) at maximum speed, for 2 min then passed a 30‐mesh sieve (0.595 mm or 595 µm, Gilson Co., Inc.). Herein, free‐flowing refers to a matrix that is predominately soluble without clumps or gelled materials, which pass a 30‐mesh sieve, flowing and pouring freely. Thereafter, samples were gelatinized in a shaker incubator (Max‐Q 6000, Thermo Scientific), at 80°C at 100 rpm. Then, saccharification occurred using food‐grade enzymes and samples were passed through a 140‐mesh sieve (0.105 mm or 105 µm, Gilson Co., Inc.) (Table 2 and Figure 2). Softening time in nongerminated BRR and WR was evaluated as controls, compared against the “optimized” GBR softening protocol. Processing losses were evaluated and calculated for: wet milling as percentage weight (g) lost on the 30‐mesh sieve per total volume (ml), as percentage weight (g) lost on the 140‐mesh sieve in 600 ml (wt) aliquots removed for enzyme treatments, and total (g) losses in all processing stages (including equipment transfers and sieve losses) per total volume (ml).

### 2.6 Saccharification enzyme treatments

Based on methodologies elaborated in related patents (Bartocci & De Luigi, 2000; Mitchell & Mitchell, 2010; Mitchell et al., 1988), initial saccharification was achieved using α‐amylase (EC 3.2.1.1) and glucoamylase (EC 3.2.1.3) at concentrations within or slightly above ranges previously described for various rice:water ratios. After gelatinization, samples were allowed to cool to approximately 55°C, and α‐amylase (BAN 480 L, CAS# 9000‐90‐2, Novozymes) added, followed by glucoamylase (GA400L, CAS# 9032‐08‐0, Amano Enzymes) within 1 hr; at 300 µl/100 g starch (Table 2, Figure 2). Brix and pH were monitored 30 min–1 hr after applying BAN, then 1.5–2 hr after applying the glucoamylase, and periodically thereafter (24 hr to 3 days) in rice beverage stored at 4°C in glass bottles (250‐ml Corning).

### 2.7 Comparison of beverage product properties

Brix, pH, and color were assessed in the rice beverages and commercially available products. Commercial rice beverages (CRB) representing four different brand names and products were purchased and analyzed for comparison. Brix (total soluble solids) was measured with a refractometer (Atago Pocket PAL‐1). pH was measured with a handheld probe (pHTestr 20, Oakton Instruments) that was calibrated routinely against a pH meter (Thermo Scientific, Orion Star A215). Color was measured by placing 15‐ml slurry in a glass 20‐ml Petri dish, with a Konica Minolta CR400 Chroma Meter. Hue was calculated using a spreadsheet formula “=DEGREES(ATAN2(a*,b*))” and if either quadrant containing the a* or b* was negative, the correction “=IF(result cell < 0,value + 360, result cell)” was applied.

### 2.8 Starch properties by rapid visco analyzer (RVA)

Gelling characteristics of commercial precooked and regular rice flours and Rondo flours produced in‐house were assessed using a rapid visco analyzer (RVA) to purposely overestimate gelatinization temperature, based on pasting temperature. WR and BRR rice samples were freeze‐dried (Virtis Genesis, 25ES, Pilot Lyophilizer, SP Industries Company) and milled into flours using an UDY cyclone sample mill (3010‐080P) with a 1.0 mm screen. BRR flours were purposely preheated in a water bath at 75 and 85°C to visualize differences in RVA profiles, compared to various WR and BRR samples. Commercial white flours used for comparisons were Rivland RL‐100 (CRF1) long‐grain rice flour (Riviana Foods Inc.), Remyflo R‐500‐P (CRF2) precooked rice flour, and Remyflo R7‐150T (CRF3) high‐amylose rice flour (Remy/Beneo). Viscometric profiles and the pasting properties were evaluated using an RVA (Model Super 4, Newport Scientific), with the 7.10 RVA Rice Method, according to AACCI Method 61–02.01 (AACCI, 1999b). Viscosity was recorded in rapid visco units (RVU) and reported as centipoise (1 RVU = 12 cP).

### 2.9 Design and statistics

Experiments were conducted wherein several treatments, replicates, and subsamples were evaluated, changing one variable at a time, in a step‐wise, empirical approach. Approximately 35 independent time/temperature and weight/volume ratios using various softening and milling protocols were tested through the course of developing a germinated brown rice process (Figure 2) to attain preliminary rice beverages. Data were analyzed in JMP^®^ 13 PRO for Windows (SAS Institute Inc.), and their distributions verified by the software, which  removed data points falling outside a normal distribution. Thereafter, data were submitted to ANOVA in JMP^®^ 13 PRO for Windows. If statistically significant differences were found, means were compared against the control by Dunnett's test at *p* < .05. On those cases in which a control was not available, treatment differences were evaluated by Tukey's Kramer HSD test at *p* < .05 in JMP.

## 3 RESULTS AND DISCUSSION

The objectives of this project were to develop a processing method for producing rice based beverages that utilized well‐characterized freshly germinated brown rice (GBR) in lieu of any starting materials that were previously stabilized. Inputs were minimized using green processing technologies geared toward delivering a free‐flowing initial rice beverage containing most of the endogenous, natural health‐beneficial constituents found in brown rice. The preliminary beverages refer to a free‐flowing, mainly soluble slurry comprised of a fresh (never frozen, dried, stabilized, or preserved) rice/water and germinated rice/water liquid matrix, subjected to saccharification enzymatic steps to convert starch into smaller molecular weight compounds and oligosaccharide starches and sugars, ready for pasteurization.

Rondo was chosen because this variety has superior blast disease resistance and agronomic production (Yan & McClung, 2010), but due to poor milling quality, it often has excessive brokens that are used in brewing and rice beverage formulations. The percentage of freshly dehulled BRR product weight attained from roughly 51.8 kg paddy rice with in‐house dehulling and culling in six assessed independent trials was 77.3 ± 6.5%, which represents an initial loss (mainly hulls) of 22.1%. Herein, we report several conditions and parameters measured to assess the efficiency and efficacy of the processing stream as a basis for initial rice beverages, prior to pilot plant scale‐up.

### 3.1 Water absorption and germination

Some commercial sprouting, especially with microgreens, involves leaving seed/grain hulls intact, then subsequent washing which may or may not be followed by hull removal and drying prior to sales (e.g., microgreens), or “stopping” the process (e.g., making a flour or freeze‐dried product). Therefore, we initially studied the soaking/sprouting process with Rondo paddy rice in comparison against freshly dehulled BRR. Paddy rice absorbed greater quantities of water than BRR and this was possibly due to the hulls absorbing water and/or water accumulation between the bran and hull (Figure 1). Paddy rice water absorption means were significantly different (*p* < .05) at all three temperatures with 37.0%, 34.9%, and 31.4% at 35, 30, and 20°C, respectively. Similarly, BRR germination temperature means were significantly different (*p* < .05) but seeds at 35 and 30° behaved roughly the same (35.1 and 34.6%, respectively) versus those at 20°C (32.9%). In general, the rate of water absorption increased with increasing germination temperature and plateaued at approximately 48 hr in BRR (37.5%–35.9%), and plateaued or slightly increased in paddy rice (41.3%–41.0%) as illustrated in Figure 1. Paddy rice which would require additional processing to remove hulls was not used further. Similar results have been observed in experiments geared toward optimizing wet‐milling conditions so as to not affect starch properties and gelatinization (Chiang & Yeh, 2002), and in brown rice (Cao, Jia, Han, Liu, & Zhang, 2015). In 30 replicates from five separate trials where 600 g BRR was germinated, there was an average percentage water (weight) gain of 40.19 ± 1.45%.

Using lower temperatures and/or longer germination duration occasionally led to off‐odor or microbial issues. Furthermore, between 48 and 72 hr germination, water (weight) loss started occurring in both rough rice and BRR. This was pronounced in paddy rice at 35°C (Figure 1a) and dehulled materials at 30 and at 35°C (Figure 1b). Catabolic loss presumably attributed to 72 hr weight loss. If germination continued, mobilization and conversion of excessive amounts of carbohydrate and protein reserves would effectively lose marketable product mass, including quality and health‐beneficial attributes. An elevated temperature for sprouting (35°C) was ultimately selected, and only 48 hr was required to efficiently deliver 2–3 mm length sprouts.

Attaining a visible sprout was also a criterion for stopping germination, similar to 0.5–1.0 mm of (Cao et al., 2015). However, the stopping point was also gauged by the aforementioned weight change plateau and macronutrient changes (Table 1). Currently, there appears to be no industry‐wide accepted or legal definition for a sprout. In 2008, the American Association of Cereal Chemists (AACC) International indicated that “Malted or sprouted grains containing all of the original bran, germ, and endosperm shall be considered whole grains as long as sprout growth does not exceed kernel length and nutrient values have not diminished. These grains should be labeled as malted or sprouted whole grain” (AACCI, 2008). However, this subjective criterion varies by seed size and has no defined relationship to nutrients. Subsequently, coleoptile length was assessed to determine approximately when, in relation to weight and observed proximate analyses changes, the sprouting process should be terminated. When considering evaluations over several independent trials, the average sprouted coleoptile length in Rondo GBR was 2.24 ± 0.83 mm (*n* = 160).

### 3.2 Food sanitation rinses and microbial assessments

Sprouts and sprouting (mainly mung bean) are regulated by the FDA criteria (Code of Federal Regulations, Title 21; Parts 11, 16, and 112), with specific requirements in subpart M (FDA, 2015). These commodities are germinated under warm, humid conditions and are classified as “fresh cuts” due to possible pathogen contamination. Subsequently, GMPs and HACCP were considered and peracetic acid and water rinses were performed to decrease microbial loads during some trials and to ascertain whether the rice kernel germination and key physicochemical parameters would be negatively affected by a robust food‐safety treatment. The percentage BRR germination in combined trials involving peracetic acid and water rinses (*n* = 200 kernels) was 97.0 ± 1.4, 98.5 ± 0.7, and 99.5 ± 0.7% for water, 30 ppm and 300 ppm peracetic acid, respectively. The percent germination of BRR in six control trials not involving peracetic acid rinsing was 96.7 ± 0.8% (*n* = 600 kernels). In combined trials involving peracetic acid and water rinses (*n* = 40 kernels), the coleoptile lengths were 2.27 ± 0.94, 2.26 ± 0.73, and 2.23 ± 0.67 for water, 30 and 300 ppm peracetic acid, respectively, indicating no significant difference in germination characteristics between the controls and peracetic acid rinses. Rinsing BRR (30 min) with deionized water decreased significantly TPC and yeast (Table 3). The opposite was observed concerning what happened to the GBR upon termination whereby all rinsing treatments had greater TPC counts and even the test maximum (570,000). Water rinses and both 30 and 300 ppm peracetic acid rinses decreased significantly molds, yet had elevated TPC and significantly greater yeast compared to the BRR control (Table 3). The germination process (post 48 hr) resulted in markedly increased TPC and yeast presence in GBR compared to BRR. Similar trends were observed in alfalfa sprouts where chlorine reduced microbial populations in seeds but TPC and coliform counts increased during sprouting (Soylemez, Brashears, Smith, & Cuppett, 2001). Both peracetic acid rinses had no effect on TPC but significantly reduced molds and yeast in the GBR compared to the BRR rinsed samples (Table 3). However, further processing revealed that the 300 ppm peracetic acid treatment was harmful to the seeds (acidity led to off‐odors and eventual decay) and it was not pursued further (data not shown).

**Table 3:** Microbial evaluation of dehulled brown Rondo rice (BRR) and germinated brown rice (GBR) before (control) and after rinsing, using 30 and 300 ppm peracetic acid

Rice type^a^	Treatment	Aerobic plate^b^ count (cfu/g)	Molds (cfu/g)	Yeast (cfu/g)^b^
BRR	Control	387,778	360	4,257
	Rinsed	160,000*^c^	212	19,108*
	GBR	399,167	130*	24,500*
	30‐PA GBR	570,000	70*	27,000*
	300‐PA GBR	570,000	10*	26,000*
WR		653	9,033	0

^a^ BRR control is the brown Rondo rice, which was water rinsed (BRR rinsed), peracetic acid rinsed (30‐PA and 30‐PA for 30 and 300 ppm), and then germinated (GBR). Rondo white rice (WR) data are provided as a comparison but were technically not the same experiment so statistics were not applied.

^b^ The minimum yeast and mold count detection limit was 10, and 570,000 the maximum TPC (aerobic plate count).

^c^ Means highlighted with an asterisk (*) are significantly different from the BRR (control) according to Dunnett's test at *p* < .05.

### 3.3 Proximate analysis and phytic acid (%)

The proximate analyses of white and brown rice (FAO and USDA) were compared against in‐house processed BRR, GBR, and milled Rondo white rice (WR). Due to intact embryo, brown rice protein is generally greater than white rice, and all BRR samples vs. WR from in‐house, FAO and USDA databases, followed this trend (Table 1). Crude protein levels in brown rice reported by the USDA and FAO were indeed greater than white rice, and our in‐house long‐grain brown rice (BRR) samples contained the highest levels (7.4%). This is due to the fact that minimal bran was removed during dehulling, keeping intact almost all the embryos, as BRR remained 96.7% viable. Furthermore, freshly dehulled rice was used and sampled immediately or it was only stored overnight at 4°C prior to use within 24 hr. The aforementioned water (weight) losses during sprouting (Figure 1) were presumed to be due to catabolism. Indeed, based on the BRR proximate analyses, apparent weight loss (water) in GBR was mostly due to solubilized and remobilized protein, fat, and carbohydrates, which along with ash, all decreased significantly in GBR relative to BRR (Table 1). Sprouting the brown rice caused reductions in crude protein (2.0%), fat (1.1%), ash (0.6%), and substantial amylolytic carbohydrate loss (21.5%). The increased moisture content in the sprouted samples and known physiologically induced endogenous enzyme system activation likewise indicates the water (weight) loss observed in Figure 1 were catabolic. Previous research also points to the fact that germination may or may not lead to certain vitamin and mineral losses (Pagand et al., 2017) but assessments were not undertaken herein. A beneficial item resulting from sprouting was significantly reduced (more than 3‐fold) phytic acid levels (Table 1) which, as the literature indicates, generally improves the digestibility and nutrient retention (Gupta, Gangoliya, & Singh, 2015; Wu et al., 2013) and even flavor (Pagand et al., 2017).

### 3.4 Processing losses and efficacy of developed method

Data were collected to ascertain percentage loss from wet milling, through passing materials past a 30‐mesh sieve and again after passing aliquots through a 140‐mesh sieve. Other losses if/when they occurred were added into these values, per replicate. Therefore, total processing percentage loss reported (Table 4) does not reflect a true mathematical summation across rows. It was anticipated that nongerminated BRR control would have the greatest processing losses. BRR had significantly greater 30‐mesh sieve loss that was also augmented after the 140‐mesh sieve (Table 4). The GBR had roughly half the processing loss compared to its nongerminated starting material (BRR). This was likely attributed to larger particle size and a lack of softening and solubilization in the BRR. BRR loss was easily observed with large amounts of gritty starch remaining on the 30‐mesh sieve. The general processing protocol (Table 2) was established to optimize GBR softening from BRR. Per Table 1, BRR contained 74.3% carbohydrates (effectively starch), which was germinated and routinely used as GBR, containing 52.8% carbohydrates. The WR samples consistently had significantly less processing loss compared to both BRR and GBR (Table 4). We attribute this to a greater starch content (Table 1, 79.0% carbohydrate) and overall smaller particle size and chemical interactions due to less fiber, protein, bran, and oil as compared with BRR (Table 1), which is discussed below. After activation of endogenous enzyme systems through germination, the GBR samples had consistently significantly less processing losses compared to nongerminated BRR controls (Table 4).

**Table 4:** Relative percentage processing loss in brown Rondo rice (BRR) that was germinated (GBR), compared to nongerminated BRR and white rice (WR)

Sample	30‐Mesh loss (%)^a^	140‐Mesh loss (%)	Total loss (%)
White Rondo (WR) control	1.45 ± 0.84 c^c^	9.70 ± 1.65 c	3.23 ± 0.85 c
Brown Rondo (BRR) control ^b^	10.92 ± 2.99 a	16.42 ± 1.19 a	15.08 ± 1.56 a
Germinated BRR (GBR)	4.30 ± 1.85 b	12.71 ± 1.92 b	8.13 ± 1.20 b

^a^ Data represent means from independent treatments where *n* = 9 (3 trials with 3 replicates each) ± standard deviation.

^b^ Note: Nongerminated BRR serves as the “control,” as BRR → GBR.

^c^ Means not connected by same letter, per groupings down columns, are significantly different according to a Tukey–Kramer HSD at *p* < .05.

### 3.5 RVA pasting appraisals

**Figure 3:**
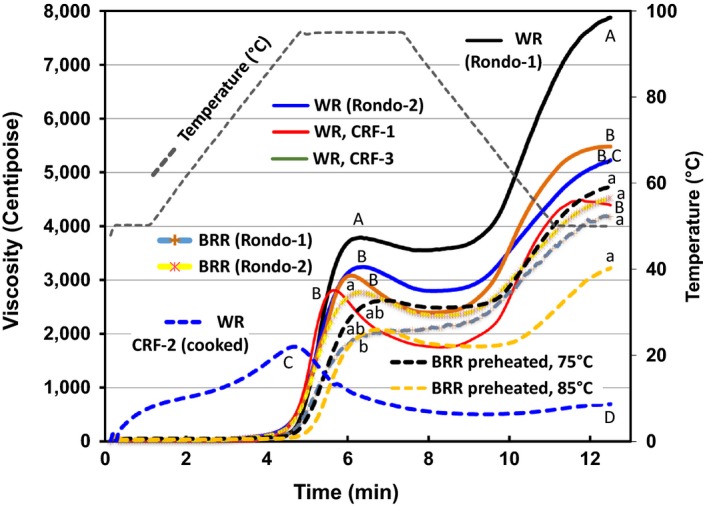
Rapid visco analyzer (RVA) profiles of brown Rondo rice (BRR) flours processed at various temperatures compared with white flour (Rondo) and commercial sample (CRF1, Rivland R100 and CRF3, and Remyflo R7‐150T) and precooked white flour (Remyflo R‐500‐P). Means (*n* = 3) not connected by same letter within each group of treatments are significantly different according to Tukey–Kramer HSD test at *p* < .05. WR sample means are designated by capital letters, and BRR sample means are designated by lower case letters

The Rondo variety is considered high amylose with an intermediate/high ratio for slowly digestible starch:resistant starch and termed a low gelatinization temperature rice (Patindol, Guraya, Champagne, Chen, & McClung, 2010; Patindol, Guraya, Champagne, & McClung, 2010). Based on the “low” WR gelatinization temperature (70°C) benchmark (Bergman, Bhattacharaya, & Ohtsubo, 2004), several different treatment regimens aimed toward fully softening the individual germinated kernels were applied, attempting to minimize gelatinization during initial stages of sieving and wet milling. Softening and wet milling were carefully evaluated in at least 15 preliminary trials, keeping temperatures near 70°C. In many of those trials, some gelatinization occurred using hot plates, certain rice:water ratios, and when wet milling was too aggressive (data not shown). These investigations, however, led to the formulation of a process whereby the softened rice and water matrix was not gelatinized, until that step was purposely performed to facilitate enzyme treatments (Table 2). Since brown rice has significantly increased levels of bran, fat, and protein compared to white rice, gelatinization temperatures should be greater, as previously reported in brown rice with various degrees of milling (Marshall, 1992) and bean flour (Carvalho et al., 2013). This was borne out by RVA pasting profiles (Figure 3), which may be used to carefully approximate starch characteristics and relative gelatinization temperatures (Dang & Bason, 2014), even though pasting temperature generally overestimates the rice gelatinization temperature (Bao, 2008). RVA profiles indicated the following: (a) the inflection point attributed to pasting temperatures were shifted to the right in all “preheated” (75 and 85°C) BRR flours, and (b) the WR and BRR control flour and two commercial WR flours (CRF‐1 and CRF‐3) behaved similarly concerning the x‐axis pasting temperature inflection points. The Rondo BRR samples had significantly lower (Tukey–Kramer HSD test at *p* < .05) pasting temperatures (87.8 and 90.7) than both preheated samples (BRR 75 and 85°C at 93.8 and 94.7°C, respectively). Furthermore, the white flours displayed the most typical starch viscosity profiles with the highest peak and final viscosities, and typical troughs and setback. The BRR preheated to 85°C had significantly lower peak and final viscosities compared to the preheated BRR at 75°C, and the highest pasting temperatures measured. The BRR RVA profiles were contrasted markedly by a precooked rice flour standard (WR, CRF‐2, Remyflo R‐500‐P) that had virtually no peak viscosity, trough, final viscosity, and, therefore, no negative setback (Figure 3). This is because a pregelatinized sample could not gelatinize again, delivering a typical starch RVA profile. The final processing protocols judiciously attempted to soften GBR using temperatures at approximately the gelatinization range previously mentioned for milled white long‐grain high‐amylose rice, like Rondo (70°C) (Bergman et al., 2004). The in‐house BRR preheated sample profiles illustrated more initial swelling compared to standards and the BRR control profiles also indicated some gelatinization potential remained in the 75°C compared to the BRR 85°C treatments, as complete gelling had not occurred.

Indeed, one could speculate based on the RVA profiles that extremely “gentle” or limited dehulling maintained a viable embryo that delivered protein, oil, and bran which increased the pasting temperature in heated BRR compared against commercial white flour standards and in‐house BRR heated samples, due to chemical interactions, as previously indicated (Carvalho et al., 2013; Marshall, 1992). Similar to the cooked white flour (CRF‐2), GBR samples delivered precooked‐like  RVA curves and profiles (essentially an almost flat baseline atop the x‐axis, far below the CRF‐2 precooked white flour), which indicated the 48 hr germination process had markedly hydrated and chemically/physically altered the starch properties (data not shown). This too subsequently allowed for GBR liquefaction protocol development at slightly higher temperatures (e.g., 75°C, max, Table 2). BRR pasting temperatures were well over the 70°C benchmark gelatinization temperature for this high‐amylose white rice, and the final viscosity characteristics indicate that significant levels of starch were present, which formed a viscous paste after cooking and cooling in the RVA but not in any GBR samples (data not shown).

### 3.6 Quality analysis of initial rice beverages and commercial samples

There were marked differences in color measurement between commercial, WR, BRR, and GBR beverages (Table 5). This was expected since white rice obviously has color removed with the bran and other chemical constituents of the embryo and aleurone. In GBR, greater a* and b* values in 30‐ & 140‐mesh loss, compared to the crude and postenzyme‐treated beverage, indicate light brown color, likely associated with bran loss. The L* and C* were significantly higher (lighter and brighter, respectively) in GBR post‐30 crude compared to their postenzyme‐treated rice beverages. Most color measures were similar for GBR and BRR. On the other hand, L* and hue were generally significantly greater in WR (Table 5). In only WR, there were no data collected for 30‐mesh loss because virtually all these samples passed the sieve.

**Table 5:** Color change in white, brown, and germinated brown Rondo rice beverages, and comparison with commercial rice beverages

Rice type	Processing step	Minolta CIE L*a*b* tri‐stimulus color									
		L*		a*		b*		C*		hue	
WR^a^	PWM crude	80.32	aA^b^	−0.94	bA	3.47	bB	3.60	bC	105.47	bA
	PWM 30‐mesh loss	–^c^		–		–		–		–	
	PNZ rice beverage	57.23	cB	−1.58	cB	0.87	cB	2.02	cB	159.83	aA
	PNZ 140‐mesh loss	60.42	bA	0.23	aC	11.91	aC	11.93	aC	89.56	cA
BRR	PWM crude	77.84	aB	−0.88	cB	6.92	cA	6.98	cA	97.25	bB
	PWM 30‐mesh loss	74.62	bA	−0.05	bB	10.31	bB	10.33	bB	90.92	cA
	PNZ rice beverage	60.54	cA	−1.96	dA	3.13	dA	3.71	dA	122.58	aB
	PNZ 140‐mesh loss	61.22	cA	0.85	aB	15.63	aB	15.66	aB	86.91	dA
GBR	PWM crude	76.58	aB	−0.80	cC	6.56	cA	6.70	cB	97.11	bB
	PWM 30‐mesh loss	62.86	bB	0.25	bA	12.29	bA	12.38	bA	92.85	cA
	PNZ rice beverage	56.41	dC	−1.84	dB	3.09	dA	3.86	dA	119.13	aC
	PNZ 140‐mesh loss	60.18	cA	1.23	aA	16.33	aA	16.38	aA	85.79	dB
GBR	CRB	53.18	b	−1.15	b	−3.49	a	3.68	b	251.48	a
BRR	CRB	65.94	a	−1.47	a	−3.71	a	3.98	a	249.08	b

Abbreviations: BRR, brown rice; CRB, commercial rice beverage; GBR, germinated brown rice; WR, white rice.

^a^ GBR CRB indicates a commercial rice beverage labeled as “sprouted” whereas BRR CRB is for commercial rice beverages labeled “brown rice.” PWM crude is postwet milled slurry, PWM‐L indicates lost materials atop the 30‐mesh sieve, PNZ is postenzyme treatment rice beverage, and PNZ‐L indicates lost materials atop the 140‐mesh sieve.

^b^ Means per rice type not connected by same lower case letter, per rice type groupings down columns, are significantly different according to a Tukey–Kramer HSD at *p* < .05. Means per treatment (processing step) that are not connected by the same upper case letter are significantly different according to Tukey's test at *p* < .05.

^c^ There was no 30‐mesh sieve loss in WR, hence no data.

Some of the most significant color difference were in b* values and the chroma (C* or total color) and hue calculations. For example, all CRB products had the lowest a* and b* values which led to the significantly highest calculated hues (Table 5). Hue angle is an attribute of a visual sensation according to which an area appears to be similar to one, or proportions of two, of the perceived colors red, yellow, green, and blue. All CRB had all negative a* and b* values whereas, aside from a few negative b* exceptions in PNZ samples, most other processed rice types (WR, BRR, and GBR) had only negative a* values. This too also caused significantly different hue means in CRB’s compared to the WR beverages, and again, compared to all in‐house GBR and BRR rice beverages (Table 5). Furthermore, commercial samples have various additives that impart physical and chemical characteristics to the products, which may also alter color.

Germinated brown rice pHs were significantly lower than BRR pHs, except PNZ after 72 hr (Table 6). Commercial products and patents indicate stabilization and fortification are often accomplished with calcium. However, the GBR samples herein have no additives and pH values were stable and close compared to CRB. After 30 to 60 min, α‐amylase and glucoamylase liquefaction resulted in ~13.7° Brix in GBR rice beverages, which increased slightly to 14.2 Brix after 2 hr (Table 6). All GBR rice beverages had very similar Brix after receiving two saccharification enzymes, and both pH and Brix had stabilized 90–120 min after enzyme treatments. pH and Brix generally increased, albeit insignificantly, three days (72 hr) into storage at 4°C (Table 6).

**Table 6:** Brix and pH change in white, brown, and germinated brown rice beverages, with commercial sample comparison

Rice type	Processing step	Time (hr)	pH	Sig^b^	Brix	Sig
WR^a^	Rinsed	0.5	5.99	b		
	PWM crude	0	6.05	a,b		
	PNZ rice beverage	0	–		15.83	a
		1.5	6.10	a	15.49	a
BRR	Water	0	7.58			
	Rinsed	0.5	7.47			
	PWM crude	0	6.47	*		
	PNZ rice beverage	0	–		14.63	b
		1.5	6.28	*	15.20	a
		72	6.34	*	15.40	a
GBR	Water	0	6.04			
	GBR‐Water	4	6.00			
	PWM crude	0	6.12			
	Gelat	0	6.04		3.08	d
	PNZ rice beverage	0	–		–	
		1	6.02		13.70	c
		1.5	6.07		14.60	a,b
		2	5.99		14.15	b,c
		24	6.08		14.83	a,b
		72	6.13	*	15.09	a
BRR	CRB #1		6.30	c	10.47	a,b
GBR	CRB #2		6.77	a	5.97	c
BRR	CRB #3		6.13	d	10.80	a
BRR	CRB #4		6.36	b	9.90	b
BRR	CRB #1,3,4		6.26	b	10.38	a
GBR	CRB #2		6.77	a	5.97	b

Abbreviations: BRR, brown Rondo rice; CRB, commercial rice beverage; GBR, germinated brown rice (Rondo) beverage; WR, white rice (Rondo).

^a^ Water indicates the starting pH of deionized water. GBR‐water is after 4 hr of germination whereas “Rinsed” indicates the pH following 0.5 hr soaking/rinsing prior to germination. PWM crude is postwet milled slurry, and PNZ is postenzyme treatment, rice beverages. GBR CRB indicates a commercial rice beverage labeled as “sprouted” whereas BRR CRB are commercial rice beverages labeled “brown rice.”

^b^ Sig. Significance. Means highlighted with an asterisk (*) are significantly different from the respective control according to Dunnett's test at *p* < .05. Means not connected by same letter, per groupings down columns, are significantly different according to a Tukey–Kramer HSD at *p* < .05.

## 4 CONCLUSION

We have established the beginning stages regarding germinating and processing unstabilized brown rice, leading to a completely green, free‐flowing soluble matrix to deliver truly sprouted brown rice beverages. Pasting temperatures in freshly dehulled brown rice that was heat‐treated were used to gauge softening and milling temperatures, prior to gelatinization and saccharification. The GBR matrix was kept free‐flowing and soluble during softening, until gelatinization was purposely accomplished to facilitate enzyme hydrolysis. Processing losses were greatly reduced in the 30‐mesh stage but losses still occurred after the 140‐mesh sieve, which need to be addressed (*e.g.,* via improved wet milling and/or emulsification). Color differences indicate that the bran and residual embryo materials containing larger particles are not always passing through the whole process. Future analysis could evaluate reducing particle size via use of additional enzymes. As the soluble solids recovered (~15 brix) already exceed most commercial samples currently found on the market, modification of enzyme use and additional solubilization may require a dilution step. As process development continues, there also will be homogenization and pasteurization that may affect color, quality, and chemical attributes, yet offer a true comparison with commercial samples.

## AUTHOR CONTRIBUTIONS

Beaulieu developed and integrated experimental concepts combining together green technologies, sprouting/germinating and creating the free‐flowing process through an empirical trial and error approach. Beaulieu designed experiments and wrote the manuscript. Obando‐Ulloa performed the statistical analysis. Beaulieu and Reed accomplished most in‐laboratory components regarding running experiments and gathering samples. Reed performed the majority of the laboratory analyses, aside from outsourced samples. McClung provided in‐depth rice knowledge and mentoring along with invaluable advice regarding several facets of the developing work, and her Center provided most of our raw rice materials.

## ETHICAL STATEMENT

The authors declare that they have no known competing financial interests or personal relationships or conflicts of interest that could have appeared to influence the work reported in this paper. All authors have no current or past (5 years) financial, funding, employment, or institutional conflicts of interest with regard to this research. Data reported in this manuscript did not include medical studies or clinical studies that involved human participants or animals. The senior author's U.S. Federal budget was the sole source of funding promoting the research. This article is a U.S. Government work and is in the public domain in the U.S.A. Mention of a trademark or proprietary product is for identification only and does not imply a guarantee or warranty of the product by the U.S. Department of Agriculture. The U.S. Department of Agriculture prohibits discrimination in all its programs and activities on the basis of race, color, national origin, gender, religion, age, disability, political beliefs, sexual orientation, and marital or family status.
